# Revealing Public Opinion towards the COVID-19 Vaccine with Weibo Data in China: BertFDA-Based Model

**DOI:** 10.3390/ijerph192013248

**Published:** 2022-10-14

**Authors:** Jianping Zhu, Futian Weng, Muni Zhuang, Xin Lu, Xu Tan, Songjie Lin, Ruoyi Zhang

**Affiliations:** 1 National Institute for Data Science in Health and Medicine, Xiamen University, Xiamen 361005, China; 2 Data Mining Research Center, Xiamen University, Xiamen 361005, China; 3 School of Management, Xiamen University, Xiamen 361005, China; 4 School of Medicine, Xiamen University, Xiamen 361005, China; 5 College of Systems Engineering, National University of Defense Technology, Changsha 410073, China; 6 Career-Oriented Multidisciplinary Education Center, Shenzhen Institiute of Information Technology, Shenzhen 518172, China; 7 Columbia College of Art and Science, George Washington University, Washington, DC 20052, USA

**Keywords:** COVID-19 vaccine, sentiment analysis, topic model, Bert, functional data analysis

## Abstract

The COVID-19 pandemic has created unprecedented burdens on people’s health and subjective well-being. While countries around the world have established models to track and predict the affective states of COVID-19, identifying the topics of public discussion and sentiment evolution of the vaccine, particularly the differences in topics of concern between vaccine-support and vaccine-hesitant groups, remains scarce. Using social media data from the two years following the outbreak of COVID-19 (23 January 2020 to 23 January 2022), coupled with state-of-the-art natural language processing (NLP) techniques, we developed a public opinion analysis framework (BertFDA). First, using dynamic topic clustering on Weibo through the latent Dirichlet allocation (LDA) model, a total of 118 topics were generated in 24 months using 2,211,806 microblog posts. Second, by building an improved Bert pre-training model for sentiment classification, we provide evidence that public negative sentiment continued to decline in the early stages of COVID-19 vaccination. Third, by modeling and analyzing the microblog posts from the vaccine-support group and the vaccine-hesitant group, we discover that the vaccine-support group was more concerned about vaccine effectiveness and the reporting of news, reflecting greater group cohesion, whereas the vaccine-hesitant group was particularly concerned about the spread of coronavirus variants and vaccine side effects. Finally, we deployed different machine learning models to predict public opinion. Moreover, functional data analysis (FDA) is developed to build the functional sentiment curve, which can effectively capture the dynamic changes with the explicit function. This study can aid governments in developing effective interventions and education campaigns to boost vaccination rates.

## 1. Introduction

According to statistics from the National Health Commission of the People’s Republic of China, as of 20 September 2022, China has reported a total of 3.44 billion doses of COVID-19 vaccines, with over 1.3 billion persons having completed the whole immunization [1]. In 2021, China preliminarily established the national immunological barrier through mass vaccination against COVID-19 via the national system and entered the third stage of regular epidemic prevention and control [2]. Although higher vaccination rates reduce the severity of breakthrough infections, there is evidence that the efficacy of one or two doses of the vaccine is decreased after six months, and COVID-19 variants strain may evolve frequently [3]. This indicates that even if vaccination rates rise, the great majority of individuals will still be infected with COVID-19. The third dosage of vaccination, as well as the children’s vaccine, is critical in averting a pandemic comeback. The understanding of the public’s emotional reactions and willingness to receive the vaccine is critical for targeted decision making during the early stages of vaccination planning, so as to avoid vaccine hesitancy and improve the effectiveness of vaccination programs.

It is known that social media has become the major channel for people to express thoughts on COVID-19 vaccination with the emergence of the epidemic and the implementation of the lockdown policy [4]. Social media has had a tremendous impact on the public’s attitude regarding vaccination. As a result, it is critical for governments, public health officials, and policymakers to understand the potential drivers that influence public sentiment regarding COVID-19 vaccination. In research related to the COVID-19 vaccine, using social media data for academic research has become an emerging trend. Social media provides a rich volume of real-time and cost-effective content including news, events, public comments, etc., [5], which has been widely used in health-related issues and public health crises [6,7,8,9]. However, research on the COVID-19 vaccine mainly employs the classic time series analysis based on discrete observation data. The dynamic change of the emotion function is frequently ignored. Moreover, traditional approaches typically employ the moving average to smooth the noise of high-frequency emotion, which makes it impossible to accurately generate the potential random process of actual observation. Finally, despite sentiment analysis models having been broadly applied in public opinion analysis, most of them employ traditional machine learning or simple sentiment analysis tools, ignoring the rich contextual semantic information hidden in the text, which makes the results of sentiment classification deviate greatly.

To fill this gap, we developed the public opinion analysis framework based on FDA combined with the deep learning transfer model Bert. Specifically, the following questions are addressed:

(1) How do we use the deep learning algorithm to capture the profound semantic and emotional information behind the microblog posts more accurately?
(2) How do we construct an intrinsic function to describe the dynamic characteristics of emotional evolution?
(3) What quantitative measurements can be used to assess the continuity and popularity of topics?

This study provides an actionable solution for depicting and predicting the dynamic characteristics of COVID-19 vaccination hesitancy. We used two years of social media data to detect subtle changes in emotions through the deep learning transfer model and explore the changes in topics in different periods through the calculation of topic continuity and popularity. The FDA obtains data with a higher signal-to-noise ratio and more accurately constructs the intrinsic public emotion to better investigate the dynamic evolution of emotion. Finally, we forecasted the public emotional evolution and the progress of vaccination. Our findings may also provide useful insights for the promotion of other vaccinations.

## 2. Literature Review

### 2.1. Sentiment Analysis

Sentiment analysis is the field of study that analyzes people’s opinions, sentiments, evaluations, attitudes, and emotions from written language and is one of the most active research areas in natural language processing [10]. The dimensions and techniques of sentiment analysis in social media texts, which are task orientation, granularity, and methodology, were reviewed by Yue [11]. Cambria et al. [12] summarized sentiment analysis in social media texts using knowledge-based, statistical, and hybrid methods. Zhang et al. [13] reviewed sentiment analysis tasks at the document, sentence, and aspect granularities. Nevertheless, not all thoughts are expressed clearly, especially implicit emotions that require natural language understanding (NLU), such as metaphors, sarcasm, and irony [14]. Keyword-based and rule-based research methodologies are used in early sentiment categorization tasks [15,16], which require a great deal of manpower and time, so they are gradually replaced by traditional machine learning [17,18,19,20], although traditional machine learning’s growth is quite limited (such as support vector machines, naive Bayes, k-nearest neighbors, hidden Markov models, conditional random fields, multi-layer perceptrons, etc.). At present, deep learning has become the mainstream sentiment analysis. Long short-term memory (LSTM) is the most used deep learning model, which is a special form of a recurrent neural network (RNN) with the capability of handling long-term dependencies [21], and the vanishing or exploding gradient in RNNs has been effectively alleviated during data transmission. However, when it comes to longer-term dependencies, LSTM is still powerless. Therefore, Vaswani et al. proposed the Transformer, a model architecture that eschews recurrence and instead relies entirely on an attention mechanism to draw global dependencies between the input and output [22], achieving a new state-of-the-art translation quality. Subsequently, Devlin et al. improved the fine-tuning-based approaches by proposing Bidirectional Encoder Representations from Transformers (Bert), which achieved new state-of-the-art results on 11 natural language processing (NLP) tasks and pioneered the use of emotion classification [23].

Because social media text has characteristics such as short text, noise, multilingualism, metaphor, irony, and so on, many topic models have been developed to quickly mine hot themes from massive unstructured text data. Latent Dirichlet Allocation (LDA) is currently the most widely used probabilistic topic model. The application of sentiment analysis in the medical field has been faster and more extensive than ever since the outbreak and spread of the COVID-19 epidemic. Multiple factors, such as public knowledge, emotions, and personal health decisions, influence public acceptance of medical interventions involving infectious diseases and vaccines. Lyu et al. [24] used the LDA algorithm and an emotion lexicon to track topics and emotions in public discussions about the COVID-19 vaccine on Twitter. Hu et al. [5] used Twitter data to reveal the American public’s opinion of the COVID-19 vaccine from a spatiotemporal perspective. Monselise et al. [25] employed non-negative matrix factorization (NMF) to determine vaccine topics, then used VADER sentiment analysis libraries and sentence bidirectional encoder representations from transformer embeddings to identify emotional content and compared the embedding to different emotions using cosine similarity. Gbashi et al. [26] systematically scrutinized media communications (Google news headlines or snippets, and Twitter posts) using three standard computational linguistics models (i.e., TextBlob, VADER, and Word2Vec-BiLSTM) to understand the prevailing sentiments in Africa on the COVID-19 vaccine. Cruickshank et al., first examined the prevalence, dynamics, and content of websites shared in vaccination-related tweets. The research found that sharing websites is a common communication strategy, and its “bursty” pattern and inauthentic propagation strategy pose challenges to health promotion [27]. Ginosar et al., examined the content of YouTube videos shared in vaccine-related tweets before the COVID-19 vaccine rollout. The research discovered the role of cross-platform sharing of YouTube videos over Twitter as a strategy to propagate anti-vaccination messages [28].

### 2.2. Functional Data Analysis

With the advancement of information gathering and storage technology in the era of big data, the complicated emotions of the public opinion process may be depicted with high frequency, of which the data presentation form is no longer an isolated discrete point, but rather a significant continuous function feature [29]. The traditional approaches typically employ the 7-day moving average to compute the evolution trend of emotion [30,31]. In essence, this method is still dependent on a typical discrete time series analysis of public opinion and emotion, ignoring the real-time continuous transformation process of emotion from time point t-1 to time point t. On the contrary, we can examine the intrinsic function of emotion from the perspective of the function curve if we can convert the irregular, discrete, and high-frequency emotion sequence into a smooth continuous function with an internal unified perspective of the function curve. Furthermore, Functional data analysis (FDA) is a strong method for filtering data noise [32,33]. It can obtain data with a higher signal-to-noise ratio, allowing it to assess public opinion and emotion data more precisely, as well as better investigate the evolution trend of public opinion.

The concept of FDA was proposed by Ramsay [34]. As a nonparametric method, it is widely used in regression, time series analysis, and curve discrimination [35,36]. Its main advantage is that it follows the “breaking up the whole into pieces” principle of big data analysis, can handle discrete and high-frequency sequences as continuous smooth functions with an internal unified structure, and relaxes data collection structural constraints and distribution settings. In terms of temporal dimension, the function type is infinite dimensional data. Therefore, in FDA, converting infinite-dimensional data into finite-dimensional space is a significant challenge. Many studies were devoted to solving this problem. The most popular strategy for reducing dimensionality is to expand functional data on a set of bases [37]. It can be divided into fixed and data-driven bases. The fixed basis method usually adopts different basis functions depending on the problem, such as polynomial, Fourier, wavelet, and spline basis functions; the data-driven basis is usually combined with the principal component analysis method, and then a limited number of principal component bases are selected to reduce the dimension of functional data through the contribution rate. After constructing the eigenfunction of the research object, we can further discuss its velocity and acceleration, as well as describe its smooth but complex process.

## 3. Method

### 3.1. LDA Topic Extraction Optimization Model

**Figure 1:**
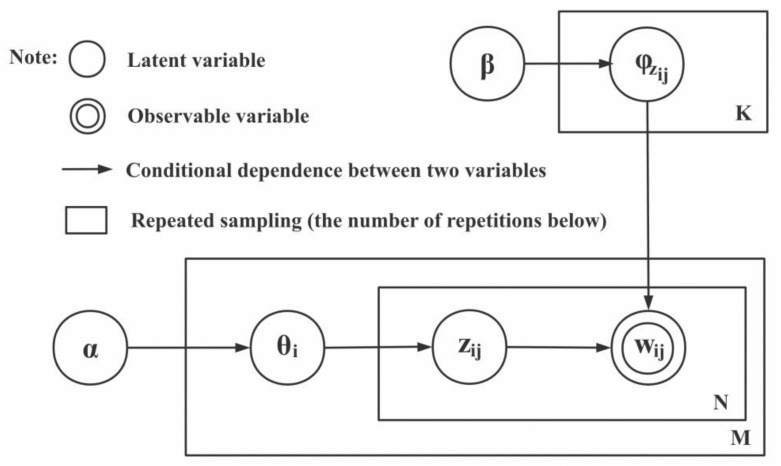
LDA topic analysis model.

To balance the computational efficiency and ability to process large datasets, we chose LDA to extract the topic. The LDA is a topic model that can provide the topic of each document in the form of a probability distribution [38]. It is a hierarchical Bayesian model with three levels, its core idea is to regard documents as a probability distribution of implicit topics and topics as a probability distribution of words. Document to topic follows a multinomial distribution, topic to word follows a multinomial distribution, and the parameters of the multinomial distribution follow a Dirichlet distribution. The modeling process is shown in Figure 1. Definite text set $D=\left\{d_{i}\left|i\in \left\{1,2,\ldots ,M\right\}\right.\right\}$ consists of *M* documents. Document $d_{i}=\left\{\left.w_{ij}\right|j\in \left\{1,2,\ldots ,N_{i}\right\}\right\}$ includes *N_i_* words, each of which corresponds to a potential topic. Then, the corresponding topic set of *d_i_* is $z_{i}=\left\{\left.z_{ij}\right|j\in \left\{1,2,\ldots ,N_{i}\right\}\right\}$. The total number of topics in document set *D* is l=$l=\overset{M}{\underset{i=1}{\sum }}$ count (*z_i_*), and the total number of words is $N=\overset{M}{\underset{i=1}{\sum }}N_{i}$. The whole document generation process is as follows:

Step 1. From the Dirichlet distribution with parameter *α*, we sample the distribution with document-topic as *θ_i_* for each document, that is, *θ_i_*~Dir (α), i ∈ 1, M, and M is the total number of documents.

Step 2. From the Dirichlet distribution with parameter β, we sample the distribution with topic-word as $\varphi_{z_{ij}}$ for each topic, that is, $\varphi_{z_{ij}}\sim \mathrm{Dir}\left(\beta \right)$, $z_{i}\in \left[1,I\right]$, and I is the total number of topics.

Step 3. For each word w_ij_ in document d_i_, we obtain a topic label $z_{ij}\sim \mathrm{Multi}\left(\theta_{i}\right)$, and generate the word $w_{ij}\sim \mathrm{Multi}\left(\varphi_{z_{ij}}\right)$.

The following is the joint distribution of all variables in the LDA model:

(1) $$P\left(w_{i},z_{i},\theta_{i},\phi \left|\alpha ,\beta \right.\right)=\overset{N_{i}}{\underset{j=1}{\prod }}P\left(w_{ij}|\varphi_{z_{ij}}\right)P\left(z_{ij}|\theta_{i}\right)\;\cdot P(\theta_{i}|\alpha )\cdot P(\phi |\beta )$$

where $P(\theta_{i}|\alpha )$ indicates the “text-topic” distribution probability of document $d_{i}$ generated by Dirichlet prior parameter *α*. $P\left(z_{ij}|\theta_{j}\right)$ denotes the *j*-th word of document $d_{i}$ generated by sampling in the corresponding topic distribution; P(ϕ|β) indicates the “topic-word” distribution matrix of topic $z_{ij}$ generated by Dirichlet prior parameter β; $P\left(w_{ij}|\varphi_{z_{ij}}\right)$ is the corresponding probability of word $w_{ij}$ generated by word distribution $\varphi_{z_{ij}}$.

The topic’s words are limited by the traditional structure of the bag-of-word model, which cannot properly incorporate semantic and contextual information. In addition, the quality of word segmentation technology has a significant impact. Therefore, this paper combined the word vector in the Bert model with the topic vector of the LDA model, obtaining the optimized topic vector *μ* through the iterative calculation of the word weight. It provided more accurate topic semantic information for the emotion simulation model of large-scale complex text.

### 3.2. Sentiment Analysis Based on Bert

Bert is a new pre-training method for language representations [22]. The semantic representation ability of the model is enhanced through the masked language model (MLM) and next sentence prediction task (NSP). Besides, it has achieved many NLP tasks depending on Transformer’s powerful feature extraction and fine-tuning transfer learning abilities.

The Bert model lacks the training of emotion corpus in the pre-training stage, which leads to its poor performance in performing emotion classification tasks. To improve the accuracy and granularity of large-scale complex text in emotion classification tasks, we developed a new pre training task for Bert and introduced an improved pre-training corpus set $TB=\left\{\left.TB_{i}\right|i\in \left\{1,2,\ldots ,M\right\}\right\}$. That is, in addition to the original Chinese Wikipedia corpus, we also added the public Sina Weibo and Baidu Tieba emotional corpus TW (https://github.com/SophonPlus/ChineseNlpCorpus), hoping that the model could learn more emotional information. At the same time, we also introduced the public Sina Weibo annotation set and a small number of emotional annotation sets of public health emergencies as the BERT in-depth pre-training corpus Finally, the final emotion classification was obtained by fine-tuning the model on the Weibo dataset $TC=\left\{\left.TC_{i}\right|i\in \left\{1,2,\ldots ,M\right\}\right\}$. The model is described as follows:

ω, δ, ρ denote token embeddings, segment embeddings, and position embeddings, respectively; *Trm* indicates the encoder unit of the transformer; $d_{i}^{’}=\{w_{ij}^{’}|j\in \left\{1,2,\ldots ,N_{i}\right\}\}$ denotes the vector set, which combines the words of the document $d_{i}$ and improved full-text semantic information.

**Figure 2:**
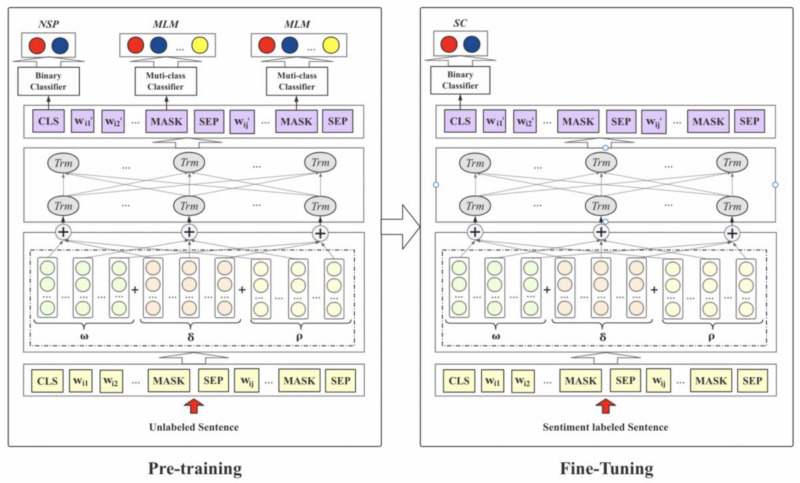
Structure of sentiment classification by Bert deep learning model.

In the pre-training phase (see Figure 2), after the segmented document $d_{i}=\left\{\left.w_{ij}\right|j\in \left\{1,2,\ldots ,N_{i}\right\}\right\}$ was input into the model, each word was mapped into three vectors $w_{ij}\left(\omega +\delta +\rho \right)$, called word embedding. The residual network structure connects the multi-head mechanism and the feed-forward layer via the transformer encoder [39]. The multi-head method calculates the attention weight by performing numerous linear transformations on the input vector.

Thus, the transformer encoder captures and stores the semantic relationship and grammatical structure information of document D. It is connected with the output layer of Softmax to adapt to transfer learning under multitasking. In this paper, we first initialize the Bert model with pre-trained parameters, and then all of the parameters were fine-tuned using labeled sentimental classification data.

### 3.3. FDA Modeling

All the m-degree polynomials constitute the m-degree polynomial space, and any group of *M* + 1 linearly independent polynomials in a polynomial space of degree *M* can be regarded as a group of bases [40]. To better reflect the regularity of complex data, the number of data transformation peaks and valleys is described by m. Through computer input and interactive modification of the fitting curve, we can achieve the goal of description. Different polynomial basis functions with different properties can be used to represent the same curve. In this paper, we selected the Bernstein basis function based on the characteristics of human–computer interaction and data mining.

**Figure 3:**
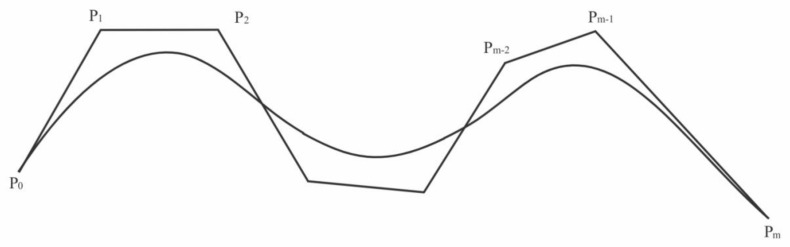
Convex hull diagram of Bernstein basis function.

Besides the good properties of normalization, symmetry, recurrence, and segmentation, the Bernstein function also has a convex-hull property. A point set’s convex hull is defined as the set of all convex combinations formed by the point set’s elements. The convex hull property of the fitting curve with the Bernstein basis function means that the curve always lies in the convex hull of its control vertex (see Figure 3).

Consider an emotional time series $Y\left(t\right),\;0\leq t\leq 1$. Let *m* − th degree Bernstein polynomials be the basis functions

(2) $$B_{j,m}\left(t\right)=C_{m}^{j}t^{j}{\left(1-t\right)}^{m-j},\;0\leq t\leq 1,\;j=0,1,\ldots ,m$$

where *C* is a combination number. The actual model can be expressed as

(3) $$Y\left(t\right)=\overset{m}{\underset{j=o}{\sum }}\beta_{j}B_{j,m}\left(t\right)+\varepsilon \left(t\right)$$

Fitting the time series data points, the sample regression equation is

(4) $$\hat{Y}\left(t\right)=\overset{m}{\underset{j=0}{\sum }}{\hat{\beta }}_{j}B_{j,m}\left(t\right)+\varepsilon \left(t\right)$$

where ${\hat{\beta }}_{j},\;j=0,1,\ldots ,m$ is the estimator of the control vertex. $B_{j,m}\left(t\right)$ denotes the Bernstein basis function. $\varepsilon \left(t\right)$ expresses the error term, that is $e\left(t\right)=Y\left(t\right)-\hat{Y}\left(t\right)$. Suppose $\varepsilon \left(t\right):\;N\left(0,\sigma^{2}\right)$ and $COV\left[\varepsilon \left(t_{1}\right),\varepsilon \left(t_{2}\right)\right]=0$ for $t_{1}\neq t_{2}$. We can further use the properties of the constructed curve to analyze the phenomenon’s development law.

In this paper, the least-squares method was utilized to estimate the control points $\beta_{j},\;j=0,1,\ldots ,m$. The time-series data $Y\left(t\right),\;i=0,1,\ldots ,n$ are first parameterized. Let $\tau_{i}$ be the indexes corresponding to $Y\left(t\right),\;i=0,1,\ldots ,n,\;\tau_{i}\geq 0$.

By normalizing the parameterization results of the above, the normalized parameterization results are generated

(5) $$t_{i}=\frac{\tau_{i}}{\max\left(\tau_{i}\right)},\;i=0,1,\ldots ,n$$

In sentiment analysis, *n* is the number of days. Then, the fitted asset price curve can be determined by the least-squares approach. We can obtain the fitted curve through the least-squares method.

(6) $$\hat{Y}\left(t_{i}\right)=\overset{m}{\underset{j=0}{\sum }}{\hat{\beta }}_{j}B_{j,m}\left(t\right),\;i=0,1,\ldots ,n$$

Furthermore, we can calculate the first and second derivatives of the emotional change curve as follows:

(7) $$y^{\prime }=\frac{\partial \hat{Y}\left(t\right)}{\partial t}=\overset{m}{\underset{j=0}{\sum }}{\hat{\beta }}_{j}\left[\frac{tm-j}{t\left(t-1\right)}\right]B_{j,m}\left(t\right)$$

The first emotional change curve can be used to describe the speed of emotional evolution.

### 3.4. BertFDA Framework for Public Opinion Analysis

**Figure 4:**
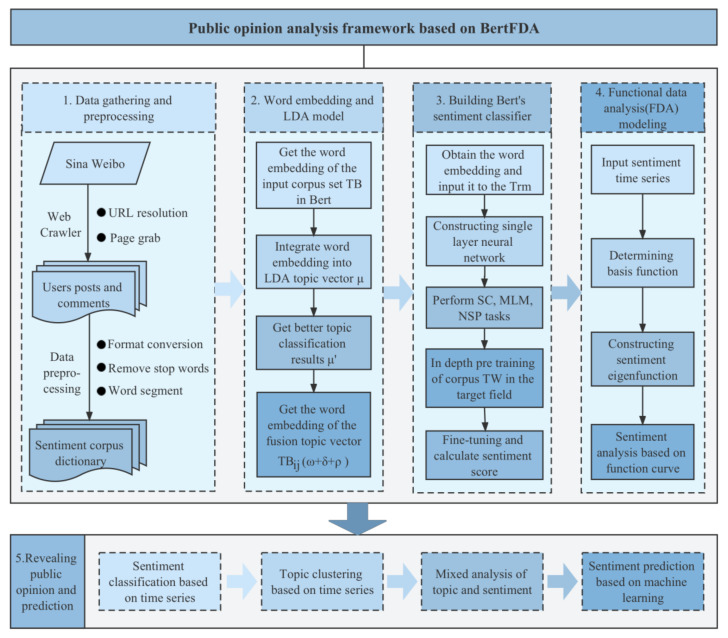
Public opinion analysis framework based on BertFDA.

BertFDA is built with the goal of accurately simulating the evolution of large-scale network public opinion, grasping the evolution characteristics and laws of groups in real-time, and assisting government departments in rapidly forming an effective public opinion response mechanism. Figure 4 illustrates the process framework based on BertFDA. The description of the algorithm framework is as follows:

Step 1: Data gathering and preprocessing: The public opinion corpus of Weibo is crawled through web crawler technology to obtain the public opinion data related to the COVID-19 vaccine. It is preprocessed by format conversion, removal of stop words, and word segmentation to form an emotional corpus dictionary, and each word corresponds to a unique index.

Step 2: Word embedding and LDA model: After inputting the corpus set TB into the Bert pre-training model, each word would be mapped to word embeddings $TB_{ij}\left(\omega +\delta +\rho \right)$. Then, it is input into the LDA model to improve the training of the topic vector *μ*. A better result *μ*′ is obtained after iterative calculation, that is, the probability distributions of *l* optimal topics and different “topic words” are derived.

Step 3: Building Bert’s sentiment classifier: The feature vector $TB_{ij}\left(\omega +\delta +\rho \right)$ output from Step 2 is introduced into the bidirectional transformer encoder, and then a single-layer neural network is constructed to connect the output vector corresponding to [CLS] in the transformer as the classifier to perform sentiment classification (SC). Simultaneously, the two pre-training tasks of MLM and NSP are retained and connected to the output vector corresponding to [MASK] and [CLS], respectively. Finally, the corpus TW is deeply pre-trained in the target field, and the COVID-19 vaccine corpus TC is finetuned to output the emotional classification and emotional value of the corpus.

Step 4: FDA modeling: Taking the emotional time series as the input, cross-validation (CV) is used to estimate the number of basis functions, and the undetermined coefficients of the model are obtained by the least-squares method. Ulteriorly, the intrinsic sentiment can be built. Finally, we obtain the sentiment evolution based on the function curve.

Step 5: Revealing public opinion and prediction: The public opinion of the COVID-19 vaccine is examined across four dimensions using the procedures outlined above: Sentiment classification and topic clustering using time series, topic emotion mixed analysis, and sentiment prediction with machine learning.

## 4. Result

### 4.1. Data Extraction and Preprocessing

Weibo has played a significant role in people’s lives as a source of information and communication. According to the Sina Weibo Data Center’s “2020 Weibo User Development Report,” the number of monthly active users peaked at 523 million in September 2020. Because Weibo contains a wealth of emotional information and popular topics, COVID-19-related microblog posts have a profound impact on the vaccination willingness of people. As a result, we selected Sina Weibo as the data source.

**Figure 5:**
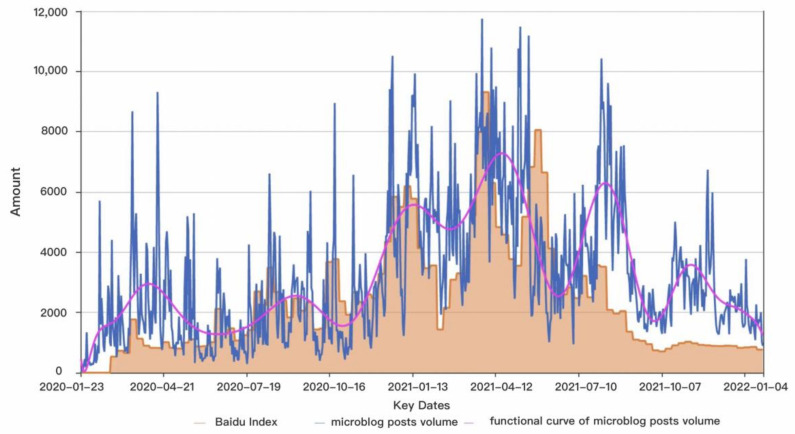
The number of Sina Weibo and Baidu index over the entire study timeline.

The Wuhan Epidemic Prevention and Control Headquarters announced the “Wuhan lockdown” on 23 January 2020. Since then, the epidemic has spread, and the number of people discussing it has gradually increased. Therefore, we built a Python-based crawler architecture using the COVID-19 outbreak as a study background and search phrases such as “new crown vaccine” and “new crown vaccination.” It collects 2,597,823 microblog posts from 0 h on 23 January 2020 to 24 h on 23 January 2022 (732 days in total). The data include the username, content, and the time of posts. As the first step in Section 3.4, the collected microblog posts are cleaned by detecting and processing duplicate and missing values, manually filtering irrelevant information such as advertisements and website links, and converting emoticons into text. For further analysis, the cleaned 2,353,435 valid microblog posts are integrated according to the time series and processed using function smoothing. In addition, we used the Baidu Index (http://index.baidu.com/, accessed on 1 October 2021) to gauge public attention. The Baidu Index is a platform based on the Baidu search engine that can integrate big data of Internet behavior and draw attention to specific keywords. As a result, we undertook an integrated analysis of public opinion in the Baidu Index throughout this study time, as well as the microblog post volume we crawled, using the search term “new crown vaccination”. Figure 5 depicts the results.

The evolution pattern of the COVID-19 vaccine in the Baidu index is highly consistent with Sina Weibo. The public debate and interest in the COVID-19 vaccine peaked between November 2020 and August 2021. Combined with specific events, the peak in October 2020 is related to the massive discussion when Pfizer revealed that their COVID-19 vaccine provides 90% protection. The spike in November 2020 is related to the vaccine being administered to a British woman for the first time. The debate increased in waves after China approved the COVID-19 vaccine. In August 2021, it was approved for usage by school pupils aged 3 to 17, and the heat of discussion was reignited.

### 4.2. Tracking Topic over Time

Tracking topics over time allows regulatory authorities to more accurately predict and control emergency risks, resulting in more efficient information services and emergency management [41]. Many researchers divide time into multiple phases to observe the dynamic evolution of topics. For example, Wang et al. [42] divide emergencies into four stages: Formation, diffusion, outbreak, and termination. In order to examine the theme of sentiment evolution at the various stages, An et al. [41] classified the event into four phases: Initial, outbreak, decline, and subside. These methods could detect topic fermentation inflection points as well as people’s overall sentiment tendencies [43]. On the other hand, most time series studies of public health emergencies are divided according to the overall situation following the event, with rather coarse time unit particles. For the COVID-19 vaccination, we want to explore the theme characteristics and evolutionary laws of microblog posts. Based on the nature of public health emergencies, it is proposed that month be used as the time unit to better observe the public opinion trend. We vectorized each month’s microblog posts to obtain feature vector representations of dimensions such as syntax, semantics, and theme, and then performed perplexity and coherence calculations on the optimized text vector to obtain the optimal number of topics, as outlined in step 2 of Section 3.4. In Table A1, we provide the results of the feature word extraction and topic distribution for a total of 24 months.

**Figure 6:**
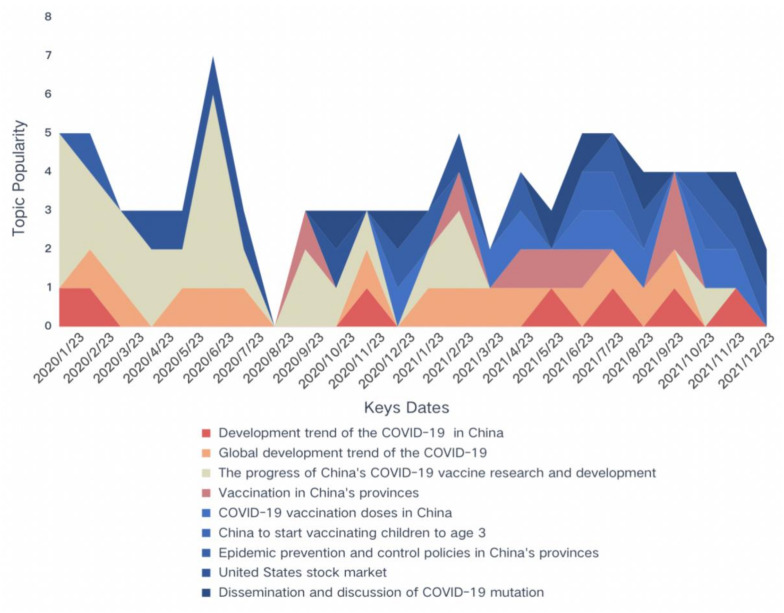
Dynamic distribution of the nine most popular topics over the entire study timeline.

The evolution of the topic can be observed by summarizing the topic feature words in each month. We obtained a total of 128 topics in 24 months. It is worth noting that nine topics appeared many times throughout the study timeline, indicating that they were frequently mentioned and discussed by the public. We mark these topics to observe the topic’s popularity. If a topic appears once a month, it is marked as 1. The more times it is marked, the higher the continuity of the topic. Accordingly, the larger the number of single marks, the higher the popularity of the topic. The dynamic distribution results are shown in Figure 6. We can observe that the topic with the highest continuity is “Global development trend of the COVID-19”, which occurred 14 times in 24 months and throughout the whole study period. The urgent need of the public for COVID-19 vaccines is closely related to the increasingly severe epidemic. Therefore, the public’s most concerning issue is the global epidemic’s development trend. The second topic is “The progress of China’s COVID-19 vaccine research and development.” It appeared 13 times, mainly in the early and middle stages of COVID-19. In terms of the popularity of the topic, its popularity in the early stages exceeded that of all other topics, especially in January and June 2020. In January 2020, the public paid special attention to the research and development plan for the COVID-19 vaccine. Good news about China’s vaccine research and development came frequently, triggering heated public discussion. For example, the Chinese Center for Disease Control and Prevention took the lead in isolating the viral strain across the world, and the first batch of vaccines developed in the Zhejiang Province of China successfully induced antibody production and entered the stage of animal trials, etc. Five months later, significant progress had been made in the research and development of multiple vaccines in China. For example, Sinopharm was the first company in the world to begin Phase III clinical trials of the COVID-19 vaccine. The world’s only COVID-19 vaccine laboratory and production workshop complex was completed in Wuhan with the strong support of the whole society and the business community. A COVID-19 vaccine by China’s Sinovac Biotech was approved for emergency use, etc. All of this positive news attracted sustained and intense attention from the public.

The topics of “Epidemic prevention and control policies in China’s provinces” and “COVID-19 vaccination doses in China” occurred nine and eight times, respectively. The similarity between the two topics is that they were mainly discussed by the public in the middle and late stages of the research period. With the increasingly mature means of preventing and controlling COVID-19 in China, all provinces issued relevant policies such as Nucleic Acid Detection, conducting source tracing, isolation and control, medical treatment, dynamic zeroing, etc., which are closely related to the public’s work and daily living. Therefore, it attracted much attention. “Vaccination in China’s provinces” related to this topic also appeared six times in the middle and late stages. In addition, with the continuous variation and spread of COVID-19, relevant discussions also appeared seven times in the middle and late stages. It should be noted that in addition to the nine topics that have been continuously discussed by the public in Figure 6, there are also some topics closely related to China’s COVID-19 vaccine. For example, the Chinese government promoted the COVID-19 vaccine to the general public. Some Chinese local governments provided free fresh eggs, vegetables, and sesame oil, Starbucks vouchers, and free tickets to parks and museums; and some local governments even subsidized monetary incentives for people who had received the COVID-19 vaccine. In addition, Olympic champion Zhang Shan became the star spokesperson of the COVID-19 vaccine promotional film, calling on everyone to get the COVID-19 vaccine. As a consequence of these encouragement and publicity measures, China’s vaccination rate for the COVID-19 vaccine rose rapidly. Therefore, the vaccination dose also became one of the topics of public concern and discussion. Another related topic is China’s vaccination reaction. The public is very concerned about the safety, effectiveness, and side effects of the vaccine. The COVID-19 vaccines approved globally have more or less side effects and adverse reactions. For example, the AstraZeneca COVID-19 vaccine may be associated with rare blood clots [44]. Although the Chinese Center for Disease Control and Prevention stated that the common adverse reactions of domestic vaccines only include dizziness, fatigue, nausea, etc., more serious cases included an allergic rash and vascular edema. Acute severe allergic reactions such as anaphylactic shock and laryngeal edema are extremely rare, and there are usually no serious consequences after timely treatment. However, such reports are still one of the reasons Chinese citizens hesitate about vaccinations.

### 4.3. Tracking Sentiment over Time

To gain a comprehensive understanding of the evolution of public sentiment during the whole research period, we constructed the Bert sentiment classifier and performed fine-tuning based on the Bert pre-training model to summarize the sentiment in all 2,353,435 microblog posts, as described in steps 3 and 4 of Section 3.4. We observed 1,962,464 positive and 392,971 negative microblog posts, accounting for 83.3% and 16.7% of all, respectively.

**Figure 7:**
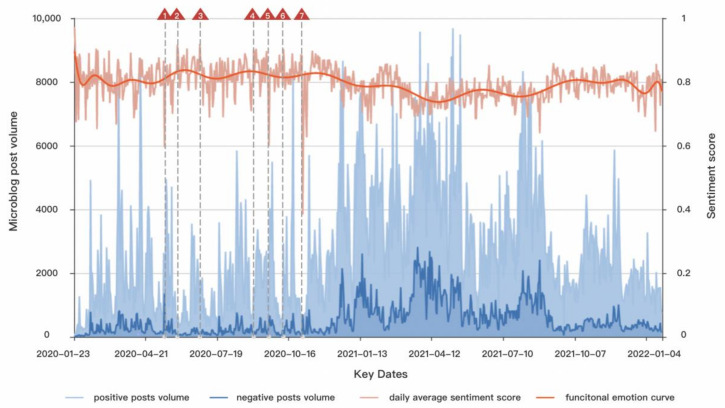
The smooth curve of average sentiment scores, and positive and negative microblog posts volume. (The red triangle represents the date with the largest fluctuation in the daily average sentiment curve).

Figure 7 depicts the smooth curve of daily average sentiment scores and positive and negative microblog post volume over a 732-day period beginning on 23 January 2020. This curve is obtained by the FDA method, where the time index is considered as the input and the original sentiment scores calculated by Bert as the output (see the orange curve). As for the number of basis functions, this is determined by minimizing the generalized cross-validation criterion [33]. We confirmed that the optimal threshold for the positive and negative sentiment was 0.61 by F1-score [45]. The daily average sentiment score and its function smooth curve show that public sentiment was mostly positive. Nonetheless, public opinion began to gradually decline after the vaccination for COVID-19 in December 2020, and as the number of vaccinations increased, so did public dissatisfaction with the vaccine, which did not improve until most citizens had completed the entire vaccination course (October 2021).

The microblog posts volume in Figure 7 shows that as the COVID-19 vaccination work progresses, the public’s positive and negative comments are increasing continuously. The cumulative number of vaccinations has gradually increased as a result of the state’s vigorous vaccination promotion. Furthermore, the Dynamic COVID-Zero Strategy in China has effectively controlled the epidemic. It may have improved public confidence and mobilized the public’s positive mood [46,47]. However, people’s willingness to be vaccinated is not as optimistic as expected in the early stages of vaccination in China. Many people are taking a wait-and-see approach, questioning the vaccine’s safety and side effects. The conclusion is consistent with reference [48]. Moreover, some people exploit a tiny number of vaccine-related adverse events to disseminate unpleasant feelings and terror across the Internet. With the ongoing mutation of COVID-19, the efficacy of vaccination has been doubted, and a growing number of people have a negative attitude toward it.

We determined the seven most volatile fluctuation dates as significant turning points in people’s sentiment based on the daily average sentiment curve, as shown by the dashed lines in Figure 6. It demonstrates that the key dates when public sentiment fluctuates the most are the early stages of the COVID-19 outbreak as well as vaccine research and development. The first stimulus occurred on 14 May 2020. We traced the contents of microblog posts that were published on this day. The World Health Organization (WHO) warned that COVID-19 may never go away [49]. As a result of the rush of harsh public remarks and the urgent need for a vaccine, public opinion sunk. On 30 May, when word spread on social media that five vaccines in China had entered Phase II clinical trials and that one inactivated vaccine might be deployed as soon as the end of 2020, public sentiment peaked. Even though public opinion changed substantially throughout vaccine research and development, overall public opinion was positive. On 27 June, the WHO said it had collaborated with several Chinese vaccine research and development teams and, by the end of 2021, the world would have received 2 billion doses of the COVID-19 vaccine. The announcement boosted public trust in the Chinese government, bringing the public mood to a new high on this day. On 2 September, Chen Wei, an academician of the Chinese Academy of Engineering, shared the development process of the COVID-19 vaccine in a large-scale public welfare program on CCTV. It greatly inspired public confidence to unite and fight the epidemic, pushing positive public opinion to a climax. On 21 September, US President Donald Trump mentioned herd immunity once more, claiming that even without a vaccine, COVID-19 would disappear. According to William Hazeltin, the top biologist in the United States, the statement was imaginary. The influence of Trump coincided with the rise in the use of HCQ (Hydroxychloroquine) across countries despite limited evidence [50]. This ineffective plan sparked American wrath and anxiety, and Chinese public opinion plummeted. Hua Chunying, a spokeswoman for the Chinese Foreign Ministry, responded on October 9 saying that China has joined the COVID-19 vaccine implementation plan and has taken practical actions to support the equitable distribution of vaccination for the world. The public voiced reassurance and confidence in the Chinese stance, boosting sentiment once more. However, it is undeniable that the contagiousness of COVID-19 is the most serious since the 1918 influenza virus. Zhang Wenhong, director of China’s National Center for Infectious Diseases, said on November 2 that it was critical to employ non-medical means to combat the epidemic before using vaccines or pharmaceuticals. This objective but cruel fact reawakened people’s dread and disquiet, causing public opinion to plummet again.

Despite the fact that the majority of people are in favor of the COVID-19 vaccine, there are still some who are skeptical. The inability to build a universal immunity barrier has been hampered by vaccine-hesitant individuals delaying or refusing to be vaccinated. On the basis of overall sentiment classification, we use the LDA to cluster 1,962,464 positive and 392,971 negative microblog posts to analyze the potential driving factors affecting public sentiment and vaccination views and compare the focus of those who have a vaccine-support attitude with those who have a vaccine-hesitant attitude. We selected the whole period for analysis to select the most significant topics by vaccine supporters and those hesitant about the vaccine for key attention and comparative analysis. We hoped that by developing a deep understanding of the thoughts and concerns among vaccine supporters, positive emotions would be guided and amplified. More importantly, we want to help the government and the media carry out targeted communication with vaccine skeptics.

**Figure 8:**
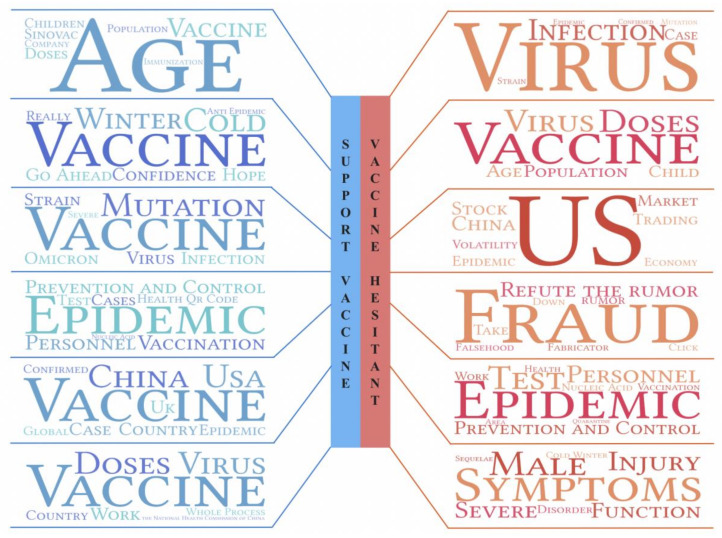
The topic keywords of vaccine-hesitant and vaccine-support groups discussion.

The most significant 12 vaccine-related topics discussed by the vaccine-supportive and vaccine-hesitant groups are shown in Table 1 and Table 2 and the word cloud of topic keywords is shown in Figure 8. Obviously, the topics discussed by the vaccine-support group are positive (topics 1 and 3) and include good wishes for overcoming the epidemic (topic 2). The vaccine-hesitant group discussed more negative news (topics 7, 9, 10, and 12). Although there are some similarities between vaccine supporters and vaccine skeptics, the emotions expressed are vastly different. For the vaccination topic, the vaccine-support group is more inclined to discuss its safety and effectiveness for children (topics 1 and 3). However, the vaccine-hesitant group is skeptical and pays more attention to the rumors and side effects (topics 8, 10, and 12). On the topic of COVID-19, the vaccine-support group is more objective and rational, and they actively cooperate with infection prevention and control (topic 5). However, the vaccine-hesitant group is more concerned about the transmission risk of variant strains such as Delta and Omicron (topic 7). It is worth noting that both the vaccine proponents and vaccine skeptics discussed the topic of “Epidemic prevention and control policies in China’s provinces” (topics 4 and 11). Although this topic is neutral, tracing the microblog posts by the two groups, we discovered that the vaccine-support group pays more attention to good deeds, and front-line workers in epidemic prevention and control. What is more, they are more tolerant and understanding of prevention and control policies in specific places during special periods. Meanwhile, some vaccine skeptics not only vent their frustrations on social media about confirmed cases, they also resist epidemic prevention efforts and try to go out with fake health QR codes. The health QR code is a personal health information and activity information tracking and judgment system invented by China for the prevention and control of COVID-19. The health QR code adopts three colors for dynamic management, among which a “green code” means you can freely pass, and the “red code” and “yellow code” mean that you must isolate yourself. Health QR codes play an important role in China’s efficient and precise epidemic prevention process. However, some people who have obtained “yellow codes” or “red codes” try to go out by copying others’ “green codes”. As a result, we discovered that public acceptance of the COVID-19 vaccine is highly correlated with the prejudgment of the epidemic and cooperation in epidemic prevention and control.

Therefore, we must foster the positive emotions of vaccine supporters while reducing the negative emotions of vaccine skeptics. We have learned that official vaccine announcements or scientific literature resonate better with the vaccine-support group on social media. On the other hand, they are more concerned with the progression of the global epidemic and vaccination. They need to know more about local epidemic prevention and control measures so they may adjust their plans in a timely manner and collaborate with relevant efforts. We need to assemble more new media resources for this group, report on more epidemic prevention and control topics, and generate more hot topics for discussion. The vaccine-hesitant group is to whom we should pay more attention and make contact. On the one hand, their unfavorable views and content on social media may convince more people, potentially impeding the orderly development of epidemic prevention and control and vaccination. On the other hand, they are the groups most likely to be persuaded to be vaccinated. Therefore, we should concentrate on the topics that concern vaccine skeptics. For example, regarding COVID-19 rumors, the media should focus on the truth and report the actual situation objectively rather than add inflammatory details to gain clicks and popularity. Simultaneously, the government should provide up-to-date information as soon as possible and combat rumors with timely, accurate, and detailed information. Regarding the topic of vaccination and its side effects in children, the Chinese government should popularize vaccination knowledge through grass-roots organizations and respond promptly to public concerns regarding the topic. It can also establish the government’s credibility and authority.

### 4.4. Sentiment Prediction Based on Machine Learning Method

The ability to obtain data and computing power has been substantially enhanced with the continuous development and innovation of a new generation of information technology represented by computers, artificial intelligence, and big data. Forecasting based on big data constitutes forming a set of mechanisms for application-based social problems. It has been published in numerous research articles, reconstructing the traditional methodological system in the field of forecasting using technical means and then providing solid theoretical and empirical research for relevant departments or institutions to achieve dynamic and accurate decision-making in major emergencies [51,52,53,54,55]. In order to reveal the essential relationship between the public and the COVID-19 vaccination more accurately and ahead of time, as well as the occurrence and evolution process, we need to employ predictive models based on machine learning and big data technology to uncover the “black box” of the vaccine-support and vaccine-hesitant groups of the public. It is critical for raising vaccination rates and mitigating the impact of a series of public health and economic crises brought on by COVID-19.

#### 4.4.1. Evaluation Criteria for Prediction Accuracy

We chose four different functions, including the mean absolute error (*MAE*), mean square error (*MSE*), root mean square error (*RMSE*), and median error (*MdE*).

(8) $$MAE=\frac{1}{N}\overset{N}{\underset{i=1}{\sum }}\left|r_{i}-\hat{r_{i}}\right|$$

(9) $$MSE=\frac{1}{n}\sum_{i=1}^{N}{\left(r_{i}-\hat{r_{i}}\right)}^{2}$$

(10) $$RMSE=\frac{1}{N}\sqrt{\overset{N}{\underset{i=1}{\sum }}\left(r_{i}-\hat{r_{i}}\right)}$$

(11) $$MdE=median\left({\left\{r_{i}-\hat{r_{i}}\right\}}_{i=1}^{N}\right)$$

where $r_{i}$ and $\hat{r_{i}}$ express the true value and predicted value, respectively.

#### 4.4.2. Experiment and Analysis

People generally care more about how well they can do in the future through sentiment-predicting approaches. Thus, the sentiment score of the previous seven days is regarded as an input variable to predict future sentiment. The original dataset is divided into two parts: A training set made up of 60% of the data and a testing set of the remaining 40%.

In this paper, the autoregressive (AR), support vector regression (SVR), random forest (RF), Gradient Boosting Decision Tree (GBDT), and Adaboost tools are considered the sentiment forecasting models. Among these, as an econometric model, AR is usually employed to solve time series problems. SVR, RF, GBDT, and Ababoost are classical machine learning models based on different theories, which are commonly used as benchmark methods of machine learning. All of the above models are executed on a Dell server with 32 GB of RAM and implemented in Python.

The determination of parameters is significant for prediction accuracy. Considering the AR model, the order is determined by minimizing the Akaike information criterion (AIC) [56]. The kener function and regularization coefficient c are selected through the grid search method. For the three tree models (RF, GBDT, and Adaboost), the most important parameters are the number of trees and the max feature. A larger number of trees would improve the performance of models, with more calculation cost. What is more, the prediction accuracy would no longer improve if the number of trees exceeds the special value. The maximum feature is determined by the features of the square root. The number of trees is optimized by cross-validation (CV), and the other parameters are gained by default values.

Table 3 shows the performances of the loss functions, such as MAE, MSE, RMSE, and MdE tests, for predictive accuracy between the different models. The SVR sentiment prediction model performs better than the other four benchmark models in terms of the evaluation criteria of the MAE, MSE, RMSE, and MdE scores, yielding values of 0.0294, 0.0014, 0.0376, and 0.0243, respectively. The Adaboost model is second only to SVR, with scores of 0.0320, 0.0017, 0.0410, and 0.0268. The results illustrate the advantages of machine learning in prediction performance.

#### 4.4.3. Predicting Sentiment over Time

**Figure 9:**
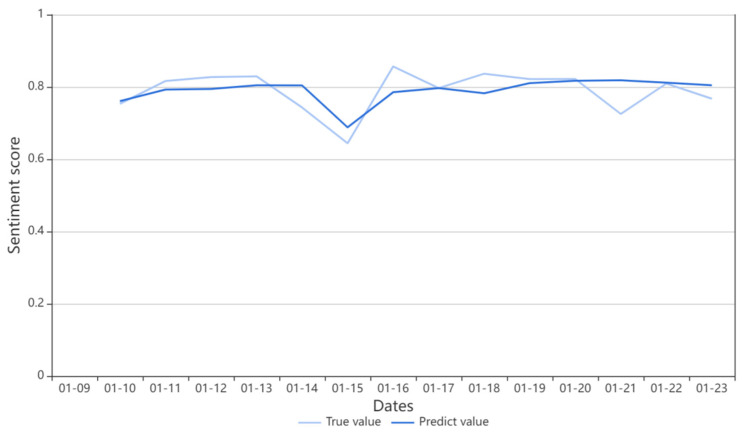
Comparisons between predicted results and true values of test data.

We use sentiment data from 23 January 2020 to 8 April 2021 (a total of 442 days) as the training set, and then input vaccine public opinion from 9 April 2021 to 24 January 2022 (a total of 290 days) into the trained model for prediction, as described in step 5 of the algorithm in Section 3.4. In order to reflect the prediction effect of the model more intuitively, we show some prediction results in Figure 9.

Figure 7 depicts the evolution of vaccine public sentiment, showing the projected and actual values from 9 January to 23 January 2022. The model accurately predicted the public’s reaction to the vaccine, which dropped until it peaked on January 15 before gradually leveling off. The average relative error between the projected curve and the actual value is less than 5%, showing that the model is quite accurate and progressing. Governments and decision-makers can prepare public opinion response plans ahead of time based on the forecast curve’s inflection points and turning points, enhancing decision-makers’ bounded rationality and governance capabilities and providing a practical and usable scientific basis for public decision-making in dealing with social issues.

## 5. Discussion

### 5.1. Principal Findings

By modeling the themes over 24 months, we discovered that the public is primarily interested in the progress and results of vaccine clinical trials, as well as the development trend and economic situation of the global epidemic, especially the US stock market. After four circuit breakers in the US stock market, it aroused heated discussions on social media. The COVID-19 vaccine’s adverse effects became a hot topic on social media during the early stages. In addition, Chinese efficient epidemic prevention measures temporarily stabilized the domestic epidemic, and those who lacked crisis awareness were unwilling to receive the COVID-19 vaccination. Certain grass-roots organizations were not appropriately guided according to the actual situation and adopted “compulsory” and “non-discriminatory” vaccination policies, resulting in the dissatisfaction of some individuals. At the same time, with the continuous mutation and spread of the new crown virus strain, people began to question the effectiveness of the COVID-19 vaccine, and there was some resistance to booster doses and the children’s vaccine.

Based on the evolution of the public’s emotions, we found that the public’s overall sentiment was more optimistic during the research and development stage of the COVID-19 vaccine, and after the official implementation of the vaccine, people’s emotions became more complicated and their expectations and concerns about the COVID-19 vaccine gradually turned into concerns and anxiety. This is owing to a significant bias in the vaccine-support group’s focus compared to the vaccine-reluctant group’s focus. In social media, the vaccine-support group is more concerned with vaccine effectiveness and the objective reporting of the news and has a more optimistic attitude towards the epidemic. The vaccine-hesitant group is more worried about the proliferation of the COVID-19 variants, the sequelae of COVID-19, and the adverse effects of the vaccine. They are more prone to believing the COVID-19 vaccination rumors. Despite the fact that some people have been vaccinated, they continue to have a negative attitude about the epidemic and refuse routine inspections. According to the BIS theory, people generate more negative emotions for self-protection [57]. Therefore, the government and public health departments should make full use of social media to understand the concerns and emotional changes of vaccine-hesitant groups on the premise of acknowledging the existence of negative emotions. At the same time, the government, as the first responsible body and leading force in the COVID-19 epidemic, should improve professionalism and accuracy in the dissemination of vaccine and vaccination information. Secondly, the government should also cooperate with social media platforms to jointly monitor and intervene in the spread of false and erroneous information and take prompt measures to stop the transmission of misinformation on social media. It can reduce the negative emotions and misconceptions of vaccine-hesitant groups. By focusing on the topics of concern to the vaccine-support group, we learned that their positive emotions are more likely to reflect group cohesion than merely personal emotions such as happiness [58]. Threats to groups, such as natural disasters or diseases, transform them into communities of interest, resulting in better conduct and social unity [59]. Therefore, the government and public health departments should pay more attention to the theme of group cohesion and develop a good connection and resonance with the public on social media to mobilize the positive emotions of vaccination supporters to a greater extent.

Through the emotion prediction model constructed in this study, we successfully predicted the public’s emotional evolution towards vaccination with an average relative error of less than 5%. According to the prediction results of the model, we found that with the popularization of the vaccine and the promotion of vaccination work, public awareness and acceptance gradually increased, but were occasionally affected by negative or false news, resulting in sharp mood swings. Therefore, the government and decision-makers can make pre-judgments, analyses, or roadshows based on the inflection and turning points in the prediction curve. It has the ability to deal with all kinds of public opinion in a short period of time and at a high rate, as well as establish the credibility and authority of the government through efficient and advanced actions.

### 5.2. Limitations and Future Work

Weibo is a popular social media platform with 523 million monthly active users, the bulk of whom are Chinese. Although it covers one-third of China’s population, it is far from representative of the public opinion of the whole of China. The time people spend on Weibo is related to age, education level, and monthly disposable income [60]. The proportion of Weibo users in the central and eastern coastal cities and provinces is significantly higher than in the western regions, and the proportion of economically developed areas is much higher than that of economically underdeveloped ones [61]. Therefore, similar to other studies that rely on social media, there may be a “digital divide” in our dataset. The study only explains how Weibo users reacted to the vaccine, ignoring the opinions of disadvantaged groups in society and those who, to some extent, remain silent on social media [5]. Secondly, due to the limitations of Weibo, we are unable to obtain all relevant microblog posts in their entirety. Weibo users are most active between 10:00 a.m. and 24:00 p.m. [62]. Public sentiment on social media can be influenced by attractive visits throughout the day [63]. As a result, our data cannot effectively proportionately shrink the distribution of different sentiments. Finally, multiple empirical studies have revealed the subtle effects of digital platform administration and control on the quality of public discussion [64]. Due to the uniqueness of the Internet environment in China, it introduces possible bias to the research [65]. While we are aware of this potential source of bias, we can assume that it only influences the overall threshold of the reported propensity for deliberation and not the comparison of different observation levels for each dimension of deliberation in the sample.

In the future, we will consider how to combine geotags of microblog posts to conduct more fine-grained sentiment analysis on users in different regions, as well as conduct investigations on a finer time scale, in order to further explore the changes in public opinion on the COVID-19 vaccine from a spatiotemporal perspective. As COVID-19 reaches the normalization stage, it will be extremely valuable if we can continue to track the evolutionary trends of public sentiment during the pandemic, account for policy considerations, and use public sentiment as a barometer of government policy performance. More importantly, the processes and algorithms utilized in this work can be extended to other similar large-scale public health crises, providing models and empirical support for governments to grasp public opinion and formulate appropriate policies.

## 6. Conclusions

Drawing on microblog posts from 23 January 2020 to 23 January 2022 (732 days in total), we examined public opinion on the COVID-19 vaccine in China. Firstly, the topic modeling of microblog posts was carried out through the improved LDA model, which effectively identified the keywords and topics discussed by the public at various stages of the COVID-19 vaccine’s development and application. Secondly, the optimized Bert language pre-training model is utilized to analyze the sentiment of microblog posts to describe the public’s emotional changes to the vaccine at various phases. Through the distinction and modeling of positive and negative microblog posts, it reveals the differences in the topics concerning vaccine-support and vaccine-hesitant groups. Finally, we use machine learning models to predict the evolutionary trends of public sentiment, providing practical significance and reference value for the government’s macro-control of COVID-19 vaccination.

## Figures and Tables

**Table 1:** The six most significant vaccine-related topics of the vaccine-supportive group (*n* = 1,962,464).

Support Vaccine		Topic Totals, *n* (%)
Topic 1	Sinovac says COVID-19 vaccine can trigger immune response in children.	233,533 (11.9)
Topic 2	Positive energy blessing about COVID-19.	192,321 (9.8)
Topic 3	Vaccines prevent severe disease from Omicron.	123,635 (6.3)
Topic 4	Epidemic prevention and control policies in China’s provinces.	107,935 (5.5)
Topic 5	Global development trend of the COVID-19.	70,648 (3.6)
Topic 6	COVID-19 vaccination doses in China.	49,061 (2.5)

**Table 2:** The six most significant vaccine-related topics of the vaccine-hesitant group (*n* = 392,971).

Vaccine Hesitant		Topic Totals, *n* (%)
Topic 7	Global transmission of new coronavirus mutation.	62,268 (16.1)
Topic 8	COVID-19 vaccination for children aged 3–11 years in China.	33,009 (8.4)
Topic 9	Stock markets in China and the United States fluctuated during the epidemic.	18,076 (4.6)
Topic 10	Rumors about the COVID-19 vaccine.	11,396 (2.9)
Topic 11	Epidemic prevention and control policies in China’s provinces.	5501 (1.4)
Topic 12	The sequelae of COVID-19 and side effects of vaccine.	4322 (1.1)

**Table 3:** Predicting results of sentiment score based on different models.

Model	MAE	MSE	RMSE	MdE
AR	0.0393	0.0024	0.0490	0.0331
SVR	0.0294	0.0014	0.0376	0.0243
RF	0.0376	0.0022	0.0472	0.0309
GBDT	0.0361	0.0021	0.0463	0.0306
Adaboost	0.0320	0.0017	0.0410	0.0268

## Data Availability

The data are not publicly available due to privacy restrictions.

## Appendix A

**Table A1:** Results of feature words extraction and topic distribution.

Time Unit	Topic ID	Topic Feature Words	Topic Label
23 January 2020 to 23 February 2020	1-1	Zhejiang, antibody, first batch, animal, experiment	The first batch of vaccines has successfully induced antibody production and entered the stage of animal test.
	1-2	Treatment, plasma, antibody, clinical, rehabilitation	Clinical trail was conducted with the initial batch of plasma.
	1-3	Research, drug, isolate, screening, virus strain	The Chinese Center for Disease Control and Prevention takes the lead in isolating the viral strain across the world.
	1-4	China, speak, come on, Wuhan, hope	Blessings of Wuhan, China.
	1-5	Research and development, advancement, route, fastest, technology	The progress of China’s COVID-19 vaccine research and development.
	1-6	Enterprises, cases, jobs, increase, counties and districts	Development trend of the COVID-19 pandemic in China.
24 February 2020 to 23 March 2020	2-1	Research and development, recombinant, academician, Wei Chen, clinical trial	The recombinant COVID-19 vaccine developed by Wei Chen’s team has been approved to clinical trials.
	2-2	Volunteer, injection, human body, experiment, first batch	The human injection experiment for China’s COVID-19 vaccine has begun.
	2-3	Work, country, test, fight the epidemic, patient	Epidemic prevention and control policies in China’s provinces.
	2-4	China, cases, confirmed, country	Development trend of the COVID-19 pandemic in China.
	2-5	United States, infection, global, United Kingdom	Global development trend of the COVID-19 pandemic.
24 March 2020 to 23 April 2020	3-1	Volunteer, Vaccination, Wuhan, Wei Chen, Phase II, Clinical Trial, Approved	Volunteers in Wuhan got the COVID-19 vaccine and took part in the phase II of clinical trials.
	3-2	Global, country, China, cases, confirmed	Global development trend of the COVID-19 pandemic.
	3-3	Animal, research, primate, clinical trial	China has announced the results of the world’s first non-human primate vaccine for the COVID-19.
24 April 2020 to 23 May 2020	4-1	United States, Trump, epidemic, president, White House	Trump claimed to have brought the White House epidemic under control.
	4-2	Antibody, report, clinical trial, no, adverse reaction	Effect of Chinese vaccine in phase II clinical trial.
	4-3	China, clinical trial, Phase II, research, team, experiment, animal	The phase II clinical trial of China’s vaccine research and availability has begun.
	4-4	U.S. stocks, rally, market, index	United States stock market
24 May 2020 to 23 June 2020	5-1	WHO, confirmed, trial, increase, United States	Global development trend of the COVID-19 pandemic.
	5-2	Inactivated vaccine, antibody, biological, China, Phase II	China has approved two inactivated COVID-19 vaccines of China-Biotics for emergency use.
	5-3	Market, United States, index, raising limit	United States stock market.
24 June 2020 to 23 July 2020	6-1	United States, Trump, WHO, global, epidemic	Global development trend of the COVID-19 pandemic.
	6-2	Sinopharm, first, clinical trial, Phase III, overseas	Sinopharm is the first company outside of China to begin Phase III clinical trials of a COVID-19 vaccine.
	6-3	Clinical trial, Wei Chen, Lancet, Antibody, Phase I	The results of phase I clinical trial of COVID-19 vaccine were published in the lancet.
	6-4	China, clinical trials, Phase II, Wei Chen, specificity	Phase II clinical trial of recombinant new crown vaccine in China.
	6-5	Production, global, workshop, Wuhan, project, completion	The world’s only COVID-19 vaccine laboratory and production workshop complex completed in Wuhan.
	6-6	Sinovac, inactivated vaccine, China, Wuhan, emergency	A COVID-19 vaccine by China’s Sinovac Biotech has been approved for emergency use.
	6-7	Mask, United States, Compulsory	The US government issued a “mask injunction.”
	6-8	Market, gold, dollar, index, rise, crude oil	United States stock market.
24 July 2020 to 23 August 2020	7-1	Russia, Vladimir Putin, country, registered	Russia registered the first Covid-19 vaccine.
	7-2	Market, gold, economy, dollar, risk	United States stock market.
	7-3	United States, Trump, response, Fauci	Fauci responds to Trump.
	7-4	Listing, forecast, month-end, price, Sinopharm	Sinopharm said the COVID-19 vaccine would ready for market by the end of December.
	7-5	Cases, strains, confirmed, India	Global development trend of the COVID-19 pandemic.
	7-6	World Health Organization (WHO), global, end, unity	The WHO hopes to end the COVID-19 epidemic within two years.
24 August 2020 to 23 September 2020	8-1	China, inactivated vaccine, emergency, United Arab Emirates (UAE)	The UAE urgently approves the use of Chinese COVID-19 vaccine.
	8-2	United States, Trump, experiment, delay	Trump, without evidence, accuses FDA of delaying coronavirus.
	8-3	Country, China, stability, relationship, Suga Yoshihide	Suga Yoshihide said stable ties with China were important.
	8-4	China, vaccine, certification, WHO, effective	WHO chief scientist said China’s new crown vaccine has been proven effective.
24 September 2020 to 23 October 2020	9-1	Adverse Reactions, Phase III, Clinical Trials, Reports, Ministry of Science and Technology	Four vaccines in China have entered phase III clinical trials, with no serious adverse reactions so far.
	9-2	Influenza vaccine, WHO, recommendations	Influenza vaccination is recommended for five groups by the WHO.
	9-3	China, Brazil, plan, volunteer	Brazilians volunteered to receive the COVID-19 vaccine from China.
	9-4	Zhejiang, Shaoxing, emergency, object, reservation	Vaccine emergency vaccination registration opens in Shaoxing, Zhejiang province, China.
24 October 2020 to 23 November 2020	10-1	United States, Trump, Melania, confirmed, White House	Trump and Melania test positive.
	10-2	Pfizer, company, Research and development, United States, effectiveness, clinical trials	Pfizer announced that the COVID-19 vaccine offers 90% protection.
	10-3	Gold, market, dollar, rebound, index, shock	United States stock market.
	10-4	Occurrence, mutation, Denmark, alert	COVID-19 mutation in Denmark.
	10-5	China, Research and development, Brazil, Ministry of Foreign Affairs, Pakistan, United Arab Emirates, progress	China’s Ministry of Foreign Affairs provides an overview of vaccine research and development.
24 November 2020 to 23 December 2020	11-1	United States, United Kingdom, years old, vaccination, Pfizer	90-year-old British woman becomes first person in world to receive Pfizer COVID-19 vaccine.
	11-2	Production, workshop, China, Lay a Foundation, first	Groundbreaking ceremony for China’s first mRNA COVID-19 vaccine production workshop.
	11-3	Confirmation, increase, accumulation, death, test	Development trend of the COVID-19 pandemic in China.
	11-4	United Kingdom, COVID-19, market, South Africa, infection, London	Global development trend of the COVID-19 pandemic.
	11-5	Adverse reaction, suggestion, crowd, emergency, response	The Chinese government has reacted to the adverse reactions to the COVID-19 vaccination.
24 December 2020 to 23 January 2021	12-1	United Kingdom, mutations, cases, deaths	COVID-19 variants identified in the UK.
	12-2	Prevention and control, test, infection, asymptomatic, nucleic acid	Epidemic prevention and control policies in China’s provinces.
	12-3	Virus, China, protection, antibody, immunity	The Chinese government is promoting the COVID-19 vaccine to the general public.
	12-4	China, economy, global, rumors, United States, Biden	The media in the US claims that China is using the opportunity to expand power.
	12-5	Vaccination, crowd, emphasis, work, reservation	COVID-19 vaccination doses in China.
24 January 2021 to 23 February 2021	13-1	Country, China, global, plan, provide, cooperation	China delivers vaccines to a number of countries in an effort to boost global anti-epidemic cooperation.
	13-2	Clinical trials, emergency, approval, inactivated vaccines	China urgently approves clinical trials of 16 COVID-19 vaccines.
	13-3	United States, global, cases, deaths, confirmed	Global development trend of the COVID-19 pandemic.
	13-4	China, first batch, arrival, aid, Sinopharm, Sinovac, Zimbabwe	Zimbabwe receives its first batch of COVID-19 vaccines from China.
	13-5	Emergency, Spring Festival, Prevention and Control, Vehicles, Health QR Code	Epidemic prevention and control policies in China’s provinces.
24 February 2021 to 23 March 2021	14-1	Research and development, China, virus, urgent, approved	China approved a recombinant protein subunit vaccine against COVID-19 for emergency use.
	14-2	Country, China, dialogue, cooperation, United States	China-U.S. high-level strategic dialogue.
	14-3	AstraZeneca, infection, research, United Kingdom, antibody	AstraZeneca vaccine causes adverse event.
	14-4	Hong Kong, Carrie Lam, citizens, vaccination, encouragement	Hong Kong’s Chief Executive encourages citizens to get vaccinated against the coronavirus.
	14-5	United States, economy, market, gold	United States stock market.
	14-6	Confirmed, cases, increase, United States, cumulative, death	Global development trend of the COVID-19 pandemic.
	14-7	China, vaccine, arrival, Sinovac, Colombia	New batch of Chinese vaccines arrives in Colombia.
	14-8	Crowd, Phase III, Adverse Reaction, Effectiveness, Situation	Phase III Clinical Trial of a China Vaccine’s Effect.
24 March 2021 to 23 April 2021	15-1	Thrombosis, Johnson & Johnson, million doses, Europe, delivery	The Johnson & Johnson Vaccine and Blood Clots.
	15-2	Immunity, mutation, antibody, infection, disease, advice	The Chinese government is promoting the COVID-19 vaccine to the general public.
	15-3	India, confirmed, increase, test, infection, nucleic acid	Global development trend of the COVID-19 pandemic.
	15-4	Global, America, forum, economy, development, Asia	The report of Boao Forum for Asia.
	15-5	Vaccine, COVID-19, Vaccination, talk, pain	China’s vaccination reaction.
	15-6	Vaccination, epidemic, reservation, Health QR Code	COVID-19 vaccination doses in China.
24 April 2021 to 23 May 2021	16-1	Vaccination, Health QR Code, prevention and control	Vaccination in China’s provinces.
	16-2	India, China, mutation, United States, virus, country	Global development trend of the COVID-19 pandemic.
	16-3	China, cumulative, ten thousand doses, cases, increase, reports	COVID-19 vaccination doses in China.
	16-4	test, prevention and control, nucleic acid, mask, personnel, district	Epidemic prevention and control policies in China’s provinces.
	16-5	Company, biology, market, sector, report	Pharmaceutical biology company annual report of China.
24 May 2021 to 23 June 2021	17-1	Vaccination, dose, first, reservation	Vaccination in China’s provinces.
	17-2	Mutation, strain, spread, India, delta	Delta variant.
	17-3	test, diagnosis, nucleic acid, prevention and control	Development trend of the COVID-19 pandemic in China.
24 June 2021 to 23 July 2021	18-1	Mutation, infection, strain, delta, expert	The delta mutation is spreading.
	18-2	China, inactivated vaccine, approved, biological, Sinopharm	China approves emergency use of the Sinopharm COVID-19 vaccines for 3-17 age group.
	18-3	Prevention and control, test, Health QR Code, nucleic acid, vaccination, district	Vaccination in China’s provinces.
	18-4	United States, cases, confirmed, epidemic, increase, United Kingdom	Global development trend of the COVID-19 pandemic.
	18-5	Vaccination, crowd, age, virus, country	COVID-19 vaccination doses in China.
24 July 2021 to 23 August 2021	19-1	Vaccine, China, Fight the epidemic, epidemic, doctor, injection	COVID-19 vaccination doses in China.
	19-2	Prevention and control, personnel, test, Health QR Code, nucleic acid, implementation	Epidemic prevention and control policies in China’s provinces.
	19-3	Virus, United States, infection, mutation, delta, case	Global development trend of the COVID-19 pandemic.
	19-4	Cases, confirmed diagnosis, test, nucleic acid, quarantine, overseas, increase, local	Development trend of the COVID-19 pandemic in China.
	19-5	Vaccination, virus, age, crowd, student	China to start vaccinating children to age 3.
24 August 2021 to 23 September 2021	20-1	Vaccination, number, virus, age, cumulative, number of times	COVID-19 vaccination doses in China.
	20-2	Mutation, inactivated vaccine, population, delta	The delta mutation is spreading.
	20-3	United States, infection, virus, Denmark	Global development trend of the COVID-19 pandemic.
	20-4	United States, veto, strengthen, plan, Pfizer	FDA advisers reject Biden’s plan to offer Pfizer boosters for all.
	20-5	Prevention and control, nucleic acid, test, quarantine, Health QR Code, personnel, masks	Epidemic prevention and control policies in China’s provinces.
24 September 2021 to 23 October 2021	21-1	Prevention and control, nucleic acid, test, Health QR Code, vaccination	Vaccination in China’s provinces.
	21-2	United States, epidemic, death, infection	Global development trend of the COVID-19 pandemic.
	21-3	Virus, booster immunization, crowd, start, focus	China launch booster shots for key groups.
	21-4	Confirmed, cases, increase, accumulation, quarantine, infect, test, positive	Development trend of the COVID-19 pandemic in China.
24 October 2021 to 23 November 2021	22-1	accumulation, Million doses, Autonomous Region	COVID-19 vaccination doses in China.
	22-2	Prevention and control, students, orderly, school, Health QR Code	China to start vaccinating children to age 3.
	22-3	Prevention and control, nucleic acid, test, mask, Health QR Code	Epidemic prevention and control policies in China’s provinces.
	22-4	age, injection, children, immunization	Vaccination of Chinese children.
	22-5	China, global, inhaled, research and development, antibody	World’s first inhaled COVID-19 vaccine introduced in China.
24 November 2021 to 23 December 2021	23-1	Cases, prevention and control, confirmed, increase, nucleic acid	Development trend of the COVID-19 pandemic in China.
	23-2	Vaccination, vaccine, injection, elderly, work, number of times	COVID-19 vaccination doses in China.
	23-3	China, vaccine, country, global, America, billion doses	COVID-19 vaccination doses around the world.
	23-4	Vaccine, Omicron, virus, mutation, strain, infection, America	The Omicron mutation is spreading.
	23-5	Masks, prevention and control, vaccination, health, region, measure	Epidemic prevention and control policies in China’s provinces.
24 December 2021 to 23 January 2022	24-1	Epidemic, prevention and control, nucleic acid, test, vaccination, Health QR Code, work, street	Epidemic prevention and control policies in China’s provinces.
	24-2	Vaccine, Novak Djokovic, tennis, international, global, rejection, crisis	Djokovic in controversy for not being vaccinated against Covid-19.
	24-3	Vaccine, vaccination, doses, virus, age, third, immunity, Wenhong Zhang	Wenhong Zhang calls for strengthening the immune barrier.
	24-4	Vaccine, infection, Omicron, cases, epidemic, mutation, America, virus, vaccination	The Omicron mutation is spreading.
